# Normal Ovariotomy

**Published:** 1874-04

**Authors:** 


Normal Ovariotomy.—Prof. T. G. Thomas, reported to the New York Obstetrical Society (Dec. 2d, 1873), a case of normal ovariotomy (removal of both ovaries as a cure of the intense and incessant nervous suffering attributable only to them, and not on account of the usual cystic degeneration) performed by him eleven days ago, which was doing very well.—*Amer. Jour. Obstet.*, Feb., 1874.

This operation was first performed by Dr. Battey of Georgia, and the above is we believe the only other operation of this kind. Dr. Battey's case is reported to have been cured.—*American Journal of Medical Sciences.*

Dr. Battey's operation has also been successfully performed by Prof. Gilmore, of Mobile, Alabama.

Dr. Battey is a fearless, bold and successful Surgeon, as those who know his surgical history for the last nine years can testify. He has been peculiarly fortunate as a Gynæcological operator, and his cures will compare favorably with the most skillful Surgeons of the day. W. T. G.

